# How does postgraduate diploma in Family Medicine impact on primary care doctors?

**DOI:** 10.1186/s12909-022-03136-w

**Published:** 2022-02-05

**Authors:** Abdul Jalil Khan, Ahsan Sethi, Sheraz Fazid, Zia Ul Haq, Javaria Raza, Mumtaz Patel

**Affiliations:** 1 Department of Family Medicine, Khyber Medical University, Peshawar, Pakistan; 2 Department of Public Health, College of Health Sciences, QU Health, Qatar University, Doha, Qatar; 3 Institute of Public Health & Social Sciences, Khyber Medical University, Peshawar, Pakistan; 4 Public Health, Institute of Public Health & Social Sciences, Khyber Medical University, Peshawar, Pakistan; 5 Department of Sociology, University of Peshawar, Peshawar, Pakistan; 6 Postgraduate Associate Dean, Health Education, England. Consultant Nephrologist, Manchester University NHS Foundation Trust, Manchester, UK

**Keywords:** Family Medicine, Family Practice, General Practitioner, Primary Care

## Abstract

**Background:**

Over the last decade, the emphasis on improving the education and training of family physicians has increased. World Health Organization has also emphasized the importance of a trained primary care workforce. In 2017, Khyber Medical University (KMU) Peshawar initiated a one-year Postgraduate Diploma in Family Medicine for doctors working in primary care, to upgrade their skills and knowledge as Family Physicians. To justify the allocation of resources, there is a need for research on the impact of such programs. This study explores the impact of Diploma in Family Medicine (DFM) on primary care doctors in Khyber Pakhtunkhwa. It also identifies the barriers associated with learning and its translation to practice.

**Methods:**

A mixed-method explanatory study was conducted from February 2019-2020. Forty-five graduates from the DFM program at KMU were invited to participate in this study. The quantitative data was collected through questionnaires (n=30) and the results were then explained further through qualitative focus group interviews (n=24). Descriptive statistics were calculated for the quantitative data and thematic analysis was performed for the qualitative data.

**Results:**

The respondents (n=30/45) were satisfied from the course content and delivery. They agreed that the course is useful (93.3%), relevant to their learning needs (86.7%) and they were able apply it to their clinical practice (100%). The qualitative findings also corroborated that the course improved both the clinical and consultation skills of the participants. The learning environment encouraged them to identify their learning needs and attain new competencies. They reported being more patient-centered and evidence-based, which increased patients’ satisfaction. The program also resulted in increased career opportunities and other monetary benefits. Despite the blended nature of the program, the participants found it challenging to balance training with the provision of services.

**Conclusion:**

One-year Postgraduate Diploma in Family Medicine is focused, practical and relevant to the learning needs of primary healthcare physicians. The policymakers should consider provision of such training opportunities in both public and private-sector. Future research should explore the long-term impact of such programs on healthcare outcomes.

## Background

Family medicine is a medical specialty that ensures comprehensive, accessible and affordable care to the community, which is necessary for the success and sustainability of any healthcare system [1] . In the developed world, a family practice approach in primary care has helped improve key health indicators [2]. During the recent Eastern Mediterranean Regional Committee's 63rd session, the World Health Organisation [3] endorsed a resolution whereby they urged member states to incorporate the Family Practice (FP) approach into primary healthcare services as a strategy for achieving universal health coverage. It was agreed that they would strengthen the capacity of their family medicine departments and train doctors working in primary healthcare, in order to achieve the goal of having three family physicians per 10,000 population by 2030. They would also adopt the WHO framework for quality improvements at the primary healthcare facilities and will implement essential health coverage [3]. Being a member state, Pakistan is also committed and has incorporated this into their National Health Vision 2016-2025 strategy.

In Pakistan, the total numbers of Primary healthcare (PHC) facilities are 11,530 [3]. There is no defined catchment population per PHC facility and no family registration or electronic medical record systems. This coupled with the lack of coordination and a formal referral system between primary, secondary and tertiary healthcare results in a system with limited continuity of care. Availability of essential health service packages is patchy and varies between health care facilities, which creates inequity in access to the services. There is more focus on curative services and hence emphasizes secondary and tertiary care. Preventive services are not integrated into the healthcare system. Even non-communicable diseases, elderly and mental health care are not part of the essential healthcare service package. The disease burden is very high, and people have minimal trust in Primary Healthcare [4].

In Pakistan, the doctors working in PHC are mostly non-specialists, with limited or no training in the delivery of primary care [1]. The concept of the trained family physicians is non-existent, as Family Medicine is not an established speciality [5]. Some of the important reasons for this under-development include little or no postgraduate training, non-existent job opportunities and poor monetary incentives leading to an outflux of new graduates towards secondary care [6]. Moreover, the undergraduate training of medical students mostly happens in tertiary hospitals with very little to no exposure to primary care, so they do not learn the required skills [4]. Lack of exposure to family medicine in undergraduate years has made it a less popular specialty amongst medical graduates in Pakistan [7].

Over the last decade, the emphasis on improving the education and training of family physicians has increased [4]. Many institutions across Pakistan have started providing accredited postgraduate training programmes in Family Medicine [8]. Recently, the Ministry of Health-Pakistan and WHO has also started a pilot project for a family practice approach across Pakistan. Two districts in each province were selected as pilot districts for the implementation of the family practice approach, and the family registration process has begun in the selected primary health care (PHC) centers. The aim is to expand this to all over the country. To achieve the target of three family physicians per 10,000 population by 2030, Pakistan will require to train more than 70,000 doctors [3].

In 2017, Khyber Medical University Peshawar started a one-year Postgraduate Diploma in Family Medicine. There was no set or agreed curriculum for the diploma family medicine in Pakistan, therefore, Kern’s Six Steps Curriculum Development approach was followed [9]. The competencies were aligned with the Royal College of General Practitioners curriculum. It focused on developing skills in advanced consultation techniques, medical teaching and leadership that are key for improving patient care. A blended learning approach was adopted, and internationally trained family medicine faculty were hired. The program is structured based on adult learning principles and has three components including online/self-reading, residential face to face sessions and placement on clinical rotations. The program enrolled non-specialist doctors working in primary care to upgrade them to Family Physicians for better service delivery. The students are allocated supervisors for both direct and indirect supervision. They are assessed through Multiple Choice Questions (MCQs), Objective Structured Clinical Examinations (OSCEs), Workplace-based Assessments (WPBA) and reflective entries in the logbooks. The course comprises six modules covering the common presentation in primary health care (Table 1). The students must complete four clinical rotations (Family Medicine, Psychiatry, Acute/Emergency Medicine, Paediatrics/Obstetrics & Gynaecology), each for two weeks duration in which Family Medicine and Psychiatry is mandatory, and they choose between the others.

**Table 1:** Outline of modules in Postgraduate Diploma in Family Medicine

Module 1	Principles of Family Medicine & Introduction to Public Health
Module 2	Non-Communicable and common chronic diseases
Module 3	Common Presentations and Emergencies in General Practice
Module 4	Common Complaints and Care of Elderly
Module 5	Women’s and Men’s Health
Module 6	Maternal & Child Health

There is a need for rigorous research on the impact of such programs to justify the allocation of resources. Hence, the current study explores the impact of this Postgraduate Diploma in Family Medicine on non-specialist doctors working in primary care. The study also identified the barriers associated with learning and its translation to practice. The findings will inform the future curriculum, policy and practices related to the development of primary healthcare facilities and the promotion of essential health coverage in developing countries.

## Methods

A mixed-method explanatory study was conducted from February 2019-2020. The study was approved by the Ethics Committee of the Khyber Medical University Peshawar.

Kirkpatrick’s four-level evaluation model was considered to evaluate Postgraduate Diploma in Family Medicine (DFM) program [10]. It is a widely used model for evaluating programs [11]. The model helped explore learners’ reactions, knowledge gained, changes in behavior and impact on the workplace [12].

Quantitative data was collected through a questionnaire. Two authors (AJK and MP) developed the initial questionnaire from the available literature. The questionnaire was reviewed by all other authors, who agreed on keeping the items limited to 10. The questionnaire was then shared with qualified medical educationists (n=4) and Family care physicians (n=3) for expert validation. Based on their feedback we included another item to the questionnaire. after the 11-item questionnaire was then piloted on 10 current students from the new cohort. The participants were responded on the items using a five-point Likert scale.. The questionnaire explored the reaction of the students to the curriculum of DFM, facilitators’ performance and application of the taught course in clinical context.

Forty-five graduates of the Postgraduate Diploma in Family Medicine (DFM) program at Khyber Medical University were invited to participate in this study and complete the questionnaire. Participants had varied clinical experience (Table 2). A participant information sheet was shared, and informed consent was taken. The questionnaire also helped recruit participants for the qualitative focus group interviews.

The focus group interviews helped explore the impact of the DFM on participants’ learning (academic performance), behavior change, on clinical practice. A total of three focus group interviews were conducted by the authors (AJK, AS and SF) in Khyber Medical University, Peshawar meeting room. Each focus group interview had 8 participants with varied characteristics (Table 2). All the interviews were audio-recorded and transcribed verbatim.

### Data Analysis

The quantitative data were analysed using STATA 14. Frequencies and percentages were calculated for demographic variables. The agreement score was calculated in percentages for each question. The qualitative data was thematically analysed. This involved reading each line, paragraph for familiarization of the data and generating in-vivo codes. Initially the two authors (AJK, AS) analyzed the data independently. The codes were identified and compared continuously with the data for coherence. The codes were then categorized into subthemes and overarching themes to accurately represent the data. These codes, subthemes and themes were then reviewed by all the authors and refined continuously to better reflect and capture coded data.

## Results

This study was conducted with students of the pioneer batch of the diploma in family medicine at Khyber Medical University, Peshawar. 30/45 students responded to the survey, while 24/30 participated in the focus group interviews. Their age range was 25 to 55 years, and all were practicing medical doctors in various health facilities of Khyber Pakhtunkhwa province. The majority of the participants were male (73.3%). Most of the doctors were working in primary care settings (70%) and had a clinical experience of over 6 years (43.33%) (Table 2).

**Table 2:** Characteristics of the study participants

Characteristics	Questionnaire Based Survey		Focus Group Interviews	
	Frequency (*n*=30)	Percentage (%)	Frequency (*n*=24)	Percentage (%)
**Age in Years**				
25-30 years	5	16.7	4	16.7
31-40 years	19	63.3	15	62.5
41 years & above	6	20	5	20.8
**Gender**				
Male	22	73.3	18	75
Female	8	26.7	6	25
**Years of clinical experience**				
<= 5 years	10	33.3	8	33.3
6 to 10 years	13	43.3	10	41.7
11 & above	7	23.3	6	25

Most of the respondents (96.7%) opined that they enjoyed the course. A vast majority (86.7%) agreed that the content of this course was focused, practical and relevant to their learning needs. All of them reported acquiring new information and knowledge during this module. The teaching methods were interesting and useful to their learning needs. The majority (90%) were of the opinion that the learning environment was friendly, venue and time schedule were convenient. All the students agreed that they had opportunities to discuss issues of interest with other participants and the facilitators. They also agreed that they were be able to utilise the knowledge and skills gained through this diploma course in their clinical practice when they were back on their jobs (Table 3).

**Table 3:** Participants satisfaction with the course

	Strongly Disagree %	Disagree %	Neutral %	Agree %	Strongly Agree %	Overall Agreement %
I enjoyed the course	0	3.3	0	6.7	90	96.7
The content of this course was focused, practical and relevant to my learning needs	0	0	13.3	23.3	63.33	86.7
I have acquired information and knowledge that is new to me	0	0	0	20	80	100
The content of course matched the learning objectives set out at start of each module	0	0	6.7	13.3	80	93.3
The learning environment was friendly, venue and timing were acceptable	0	0	10	10	80	90
The teaching methods used were interesting and useful to my learning needs	0	0	3.3	6.7	90	96.7
The facilitator encouraged our participation and questions	0	0	3.3	3.3	93.3	96.7
I had opportunities to discuss issues of interest to me with other participants and facilitator	0	0	0	10	90	100
I will be able to use the knowledge and skills gained through this course in my clinical practice	0	0	0	6.7	93.3	100
Overall usefulness of the course	0	0	6.7	3.3	90	93.3
I would recommend this course to my colleagues	0	0	3.3	3.3	93.3	96.7

### Impact

The thematic analysis of qualitative data showed that the DFM program had a positive impact on the participants, who identified gaps in their knowledge and learned new skills. The themes identified are described below:

### Improved consultation skills

The participants reported improved consultation and communication skills. They were able to identify the lack of focused listening and other observation skills. After completion of the diploma, they started practicing active listening, learned new techniques such as exploring Ideas, Concerns and Expectations, which improved their relationship with their patients.

*“Before joining this program my consultation lasted for merely 2-3 minutes, and now through reflective entry and communication skills I learned the concept of Ideas, Concerns and Expectations which we were unaware of previously” (G2P3)*

### Increased patient centeredness

They admitted that this course changed their perception of patient management. They had become more patient-centred. Now they give more time to their patients, listen to their needs, and involve them in shared decision-making for their management.

*“It [DFM course] was like a breath of fresh air because we are still practicing in the old fashion, i.e., doctors dominated consultation, but this course made us realize that we should consider patient first, involve them in all decisions and I think that changes me a lot.” (G1P4)*

They were able to identify a new doctor-patient relationship, as their consultation changed from doctor centred to patient-centred.

### Evidence-based practice

The participants learned new updated, evidence-based management guidelines and their clinical knowledge improved. Moreover, they identified the importance of managing the psychosocial aspects of the patient conditions, along with managing the pathological problem. As opined by some of the doctors:

*“Before joining this program, I was not confident in managing Hypertension and Diabetes patients, but after learning the updated guidelines, I feel confident in managing both now” (G3P5)*

The course served as refresher for the doctors with an emphasis on holistic approach of patient treatment.

### Improved patient satisfaction

The participants mentioned that implementing what they had learned during the course resulted in improved patients' satisfaction. Some of the participants were of the view that patients prefer to consult them instead of other colleagues. Their seniors and managers also appreciated them due to the positive feedback by the patients regarding improved consultation skills.

*“Patients have increased faith in me now ... Patients come back for follow up and specifically ask for me in my duty hours” (G2P1)*

### Increased career opportunities

The participants were very optimistic about increased career opportunities after completion of this course. They considered the lack of family medicine specialists in Pakistan, giving them an edge for getting better jobs. They added that it would also increase their job opportunities in the Gulf countries, so the prospects are good even if the family practice approach does not get implemented in Pakistan.

*“If we do not get opportunities in Pakistan, we will get better opportunities abroad specially in UAE” (G3P5)*

### Monetary benefits

The patients flow to their private clinics has increased. They also expect to receive some financial incentives from the government.

*“In our clinics' the number of patients has increased, and more patients are getting benefit” (G2P4)*

### Development of Primary Healthcare

The participants agreed that they are now able to provide comprehensive primary care services to the patients at their doorsteps instead of referring them unnecessarily to the far-flung secondary and tertiary level as per the conventional practice in Pakistan. This is a cost-effective approach and reduces the burden on health services.

*“This will reduce the burden on tertiary care hospitals, and we will provide some relief to the patients at their doorsteps” (G1P4)*

### Barriers

The students also shared their views about obstacles/barriers to their learning during this course and its application in the workplace. Some of them are elaborated by the respondents as under:

### Balancing multiple roles

Most of the participants were full time practicing doctors at different health units of Khyber Pakhtunkhwa so had issues with time management. It was difficult for them to maintain the balance between the contact session and their clinical work because of the increased workload at their workplace so some had to skip their private practice.

“*We all work in other areas and cities, managing this course with our jobs and family life, can be difficult” (G2P1)*

### Inability to adapt with technology

Some participants were not familiar with online learning, the use of learning management system, computer, and other software.

*“I am very weak in the management of online academic activities, and it was difficult for me to use any software" (G1P7)*

For some of the participants it was the first time they utilised such facilities.

### Insecurity among colleagues

Due to increased patient satisfaction, the participants added that they felt some insecurity among their seniors, who felt threatened by those doing this course. The management and administrative staff also resisted implementing any changes and encouraged practicing the conventions.

*“Some senior doctors do not want this program to be successful as they feel threatened and afraid of their practice being affected” (G1P4)*

### Lack of patients’ education

They noted a lack of patients’ understanding regarding the provision of standard care and clinical practice. The participants had the opinion that patients also need orientation to the ideal consultation methods. Some patients preferred quicker consultation and got annoyed due to longer appointment times.

“*Patients are not oriented with being treated differently as they are used to old style consultation*” *(G1P6)*

## Discussion

Participants were satisfied with the course and found the content, focused, practical and relevant to their learning needs. They agreed that they had acquired new competencies and implemented them in their clinical practice. Our results were in line with other studies on various programmes in family medicine worldwide [13] [14]. A study conducted at centre for postgraduate studies in Family Medicine, Saudi Arabia, evaluated their diploma programme using Dundee ready Education Environment (DREEM). It showed that the overall learning environment was right, including encouragement by faculty, but students were critical of the teaching methods, and 50 % rated it negatively. Their sample size was only ten students, amongst which five scored low. However, 80% of their students reported on their learning as going in the right direction [15]. Another study on evaluating Saudi family medicine training noted overall satisfaction with teaching methods and achieving desired learning objectives, but 25.8 % of students did not find their teachers as helpful supervisors [16]. In our study, 96.67 % of students found the teaching methods interesting and useful to their learning needs which were likely due to our experienced and approachable faculty. However, in the qualitative section of our study, some students found technology as a barrier to learning.

Participants of our study reported that their consultation skills significantly improved, which they implemented this in their clinical practice. A study performed in Hong Kong found similar results where students noted an improvement in consultation and practice organization skills after attending their DFM postgraduate course. They appreciated the sections on communication skills [17]. This also matched with the results of Al-khathami, while evaluating Saudi Family Medicine training, in which the students also acquired new presentation and communication skills [16].

Our study reported significant behaviour changes in trainees. They knowledge and skills they learned are applicable and have made positive changes in their clinical practice. They have reported a better relationship with their patients and now using a holistic patient-centered approach. They all mentioned that they give more time to the patients, explore their ICE and involve them in decision making. Now they use standardized clinical management guidelines which improve patients' care.

A study in Laos while evaluating the CPD programme for primary care physicians noted a significant increase in performance by implementing the gained knowledge in their clinical practice. They have seen improvement in all aspects of care including consultation, procedural and diagnostic skills. They were able to strengthen the capacity of staff by training their colleagues [13]. Evaluation of Saudi family medicine training also noted their trainees, adopting a patient-centred approach with effective consultation, after joining their course. They reported providing better services by adopting family medicine principles. They were more confident in dealing with chronic disease management, similar to our study. It helped in improved patient satisfaction and a better health outcome [16]. Another similar Study in Saudi Arabia, evaluating the Saudi Diploma Family Medicine program noted that FM concepts and special consultation skills had developed trainees into competent Family physicians [18]. It is similar to some comments from participants of our study, where they admit not being aware of these concepts and skills before and reforming their practice by adopting them. Their approach towards patients become more patient-centered and holistic. They also became more self-directed and research-oriented learners and used critical analysis for lifelong learning similar to our trainees now adopting reflective learning.

The study evaluating the diploma family medicine course in Hong Kong noted significant changes in the participants’ practice, including better documentation and teamwork. Some also managed to do changes in their management system by creating an appointment system and offering extra time for counselling. They also found a change in the clinical practice of their participants as they were able to deal with more complex problems and started considering the psychosocial factors along with the patient’s physical problems [17].

The participants of our study were quite convinced with improving the standard of care through the family practice approach. They noted an increase in patient satisfaction and hence an increase in patient flow. They consider this as a very positive effect as most of them noted an increase in their private practice. They were also very optimistic about increased career opportunities due to completing this course even though family medicine is not an established speciality in Pakistan. They consider the lack of family medicine specialists in Pakistan, giving them an edge for getting better jobs in future. They were still positive even if the family practice approach does not get implemented in Pakistan due to career prospects in Gulf countries.

Yoon et al. noted an increase in the number of patients attending the hospitals after starting the CPD programmes for their primary care doctors. The facilities also got renovated to catch up with improved medical practice. They found an improved relationship between the healthcare facilities. They have not checked if there were any incentives for the doctors [13]. In our study, we can still not see if it had made any effect on the relationship with other step-up healthcare facilities as there is no formal referral system in place. However, some participants commented that some of their managers had appreciated the improvement in the standard of care. They all agreed that the family practice approach is crucial, for providing quality services at primary care and will reduce the burden on the tertiary care hospital, which is a significant challenge in a country like Pakistan. Al-khathami, also argued the importance of quality health care at the primary care level for patient satisfaction and better health outcomes [16]. The SWOT Analysis of Family medicine programme in Iran identified that implementation of the family medicine program is essential to improve and strengthen primary health care in a developing country like Iran in a very cost-effective way [19]. The Besrour papers on developing family practice also reported that the implementation of family medicine practice is the key to overcome the challenges in developing countries who are having the issue of brain drain while the community is facing enormous challenges regarding health care [20].

The participants of our course found it challenging to attend all their contact sessions due to having difficulty taking time off from their work, although they agreed that they should have had more study hours. Similar findings were noted by Al-khathami, as most of their trainees felt that the duration of their family medicine diploma needs to be revised to ensure better training [16]. Even though our participants were able to apply the consultation skills in practice, but this increased their workload and they found it challenging to apply in their practice. The participants suggested that patients also need orientation towards the new consultation approach. Some had issues when bringing any changes in the system at the workplace. Some seniors took them as a threat, and they had other issues with the management. They argued that doing fewer consultations for providing better patient care created issues for the management as they are understaffed. However, some also mentioned being appreciated.

This study was based on the graduate of one program and only included recent graduates. We believe that some feedback from other stakeholders, including faculty and patients, could have been considered. Although, the long-term impact could not be evaluated as this is a new program. Our findings can be a useful step for the decision-maker for further improvements. It will help the stakeholders, including Khyber Medical University, World Health Organisation and Department of Health (DOH) enhance the quality of the training of the doctors providing primary healthcare services in the region. The health department can make this course compulsory for primary care physicians rather than the current approach of offering them voluntarily. Moreover, the findings also help justify the funding. The DOH should support primary care doctors who wish to undertake this diploma course. To encourage and attract more trainees, the DOH should take responsibility for their training and bear their tuition fee expenses. They should be given leave for the contact sessions and clinical rotation part of their course. The government can then make it a compulsion for them to continue providing their services in primary care units as family physicians.

## Conclusion

The Postgraduate Diploma in Family Medicine offered by KMU is focused, practical and relevant to the learning needs of primary healthcare physicians in Pakistan. The learning environment encouraged the participants to identify their learning needs and learn new competencies. The program improves both the clinical and consultations skills of the participants. The participants reported becoming more patient-centred and evidence-based, which improved their patients’ satisfaction. The program also results in increased career opportunities and other monetary benefits through increased patient referrals and consultations for the doctors. Despite the blended nature of the program, the participants found it challenging to balance training with the provision of services. The study findings will help the policymakers to enhance the quality of the training and its resources through a public-private partnership. Involving the community in strategic planning will help increase awareness amongst the population on the need for family medicine. Future research needs to explore the long-term impact of such programs in family medicine on healthcare outcome.

## Data Availability

All of the data and material is owned by the authors and/or no permissions are required and can be provided upon request.
